# Correction: 14-3-3 binding to LRRK2 is disrupted by multiple Parkinson’s disease-associated mutations and regulates cytoplasmic localization

**DOI:** 10.1042/BJ20100483_COR

**Published:** 2025-06-18

**Keywords:** 14-3-3 protein, cytoplasmic localization, LRRK2, Parkinson’s disease, pathogenic mutation, phosphorylation

It has come to the attention of the authors of the article “14-3-3 binding to LRRK2 is disrupted by multiple Parkinson’s disease-associated mutations and regulates cytoplasmic localization” (DOI: 10.1042/BJ20100483) that there is a duplication within panels shown in Figure 7 - namely panel 3 (E10K mutant) and panel 36 (G2019S mutant) are the same. Additionally, the numbering of panels is incorrect within the figure following panel 36.

Raw data from this experiment have been identified and assessed, and it has been confirmed that the correct image was used for panel 3 (E10K mutant) and that the incorrect image was used for panel 36 (G2019S mutant).

The raw data and requested correction have been assessed by and agreed with the Publisher. The authors apologise for the error and any inconvenience this may have caused. The data analysis and conclusions are not affected by the error.

A corrected Figure 7 is presented here, with the correct numbering of panels.

**Figure 7:**
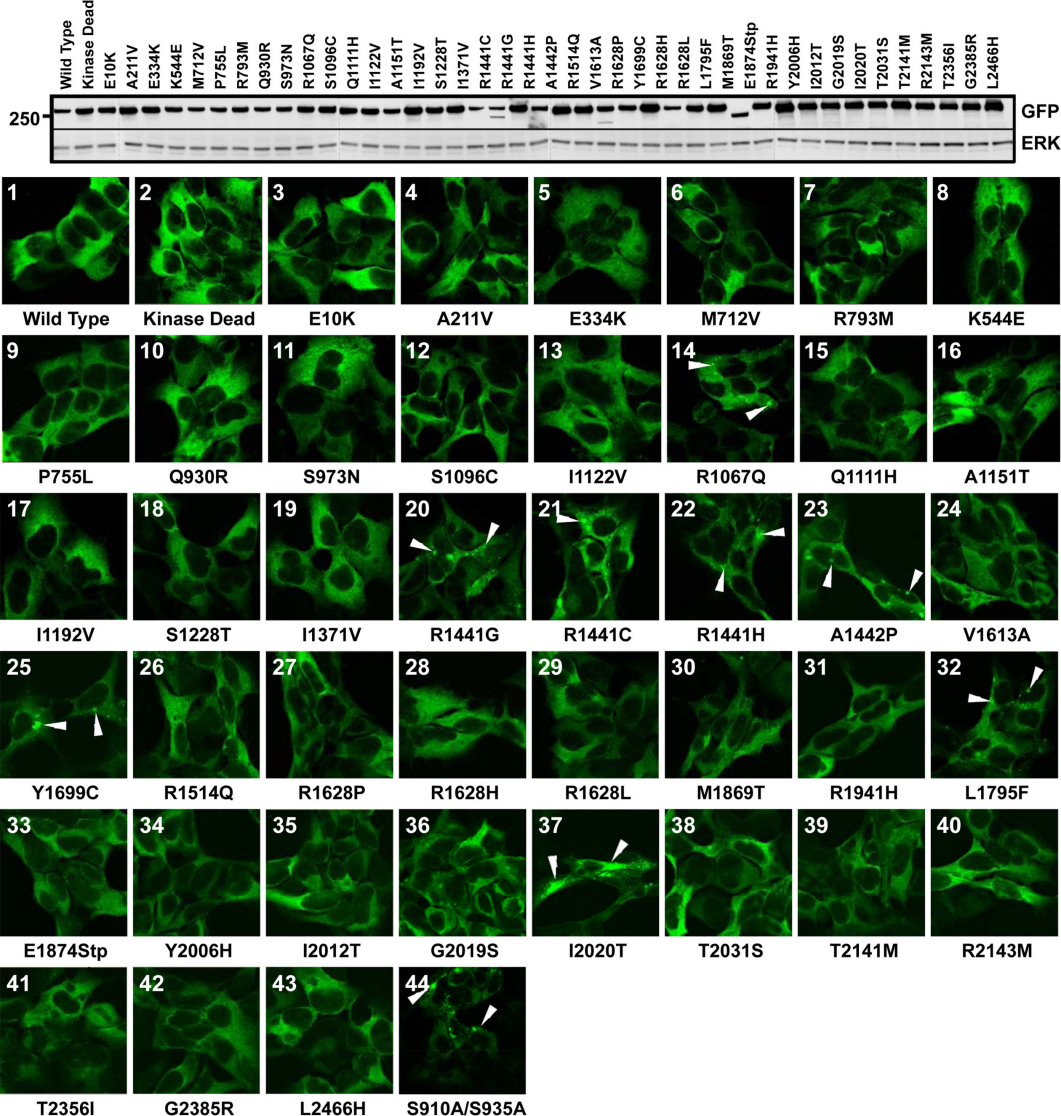
Localization of 41 PD-associated LRRK2 mutants. Parallel cultures of stable inducible T-REx cell lines harbouring the indicated mutations were induced for 24 h with 1 µg/ml doxycycline to induce expression of GFP-LRRK2. Upper panel, equal amounts of cell lysate from induced cells of each mutant were subjected to immunoblot analysis with anti-GFP antibodies to detect the fusion protein or anti-ERK1 (extracellular-signal-regulated kinase 1) antibodies as a loading control. The molecular mass is indicated on the left-hand side (kDa). Lower panel, fluorescent micrographs representative of cultures of each PD-associated mutant (panels 1–44) are shown. Cytoplasmic pools of GFP-LRRK2 are indicated with white arrowheads. Localization analyses were performed in duplicate, on two independently generated stable cell lines.

